# Antioxidant and Preventive Effects of Extract from *Nymphaea candida* Flower on *In Vitro* Immunological Liver Injury of Rat Primary Hepatocyte Cultures

**DOI:** 10.1093/ecam/nep003

**Published:** 2011-06-23

**Authors:** Jun Zhao, Tao Liu, Long Ma, Ming Yan, Zhengyi Gu, Yi Huang, Fang Xu, Yu Zhao

**Affiliations:** ^1^Department of Traditional Chinese Medicine and Natural Drug Research, College of Pharmaceutical Sciences, Zhejiang University, Hangzhou, 310058, China; ^2^Xinjiang Key Laboratory for Research and Development of Uighur Medicine, Institute of Materia Medica of Xinjiang, Urumqi, 830002, China; ^3^College of Public Health, Xinjiang Medical University, Urumqi, 830054, China

## Abstract

*Nymphaea candida* is traditional Uighur medicine that is commonly used to treat head pains, cough, hepatitis and hypertension in Xinjiang of China. In this article, the extract of *N. candida* was measured for antioxidant activity, using 1,1-diphenyl-2-picrylhydrazyl (DPPH) radicals scavenging assay and reducing power determination, and compared with those of the positive controls of butylated hydroxytoluene (BHT) and gallic acid (GA). The active extract was further purified by liquid-liquid partition to afford four fractions, of which the ethyl acetate-soluble (EA) fraction (NCE) exhibited the strongest antioxidant capacity with IC_50_ value of 12.6 *μ*g/mL for DPPH. Thirteen phenolic compounds were isolated from this fraction, and they all showed significant antioxidant activities in DPPH model system. Furthermore, NCE showed potent antioxidant capacity with IC_50_ value of 59.32 *μ*g/mL, 24.48 *μ*g/mL and 86.85 *μ*g/mL, for O_2_^−^, *·*OH and H_2_O_2_ radicals, respectively. Moreover, NCE on BCG plus LPS-induced immunological liver injury was evaluated using primary cultured rat hepatocytes. NCE produced significant hepatoprotective effects as evidenced by decreased supernatant enzyme activities (AST—aspartate transaminase, *P* <  .01; ALT—alanine transferase, *P* <  .01) and nitric oxide (NO, *P* <  .01) production. These results revealed the *in vitro* antioxidant and hepatoprotective activities of NCE against immunological liver injury. Further investigations are necessary to verify these activities *in vivo*.

## 1. Introduction

Liver is considered a key organ in the metabolism, secretion, storage and detoxifying functions in the body, and hepatic damage is associated with distortion of these functions [1]. Liver diseases are mainly caused by toxic chemicals, excess consumption of alcohol, infections and autoimmune disorders. Liver produces large amounts of oxygen free radicals (reactive oxygen species (ROS)) in the course of detoxifying xenobiotic and toxic substances, and oxidative stress caused by ROS has been shown to be linked to liver diseases, such as hepatotoxicity, and other liver pathological conditions [2, 3]. The immunological hepatotoxicity of primary cultured rat hepatocytes can be induced by Bacille Calmette-Guerin (BCG) combined lipopolysaccharide (LPS) treatment *in vitro*, and this model has implicated the involvement of release of various cytokines and active free radicals [4, 5]. Thus, immunological mechanisms and oxidative stress play important role in liver injury induced by BCG plus LPS [6]. At present, this model has frequently been used as useful experimental means for testing and developing new drugs [7–9].

**Figure 1:**
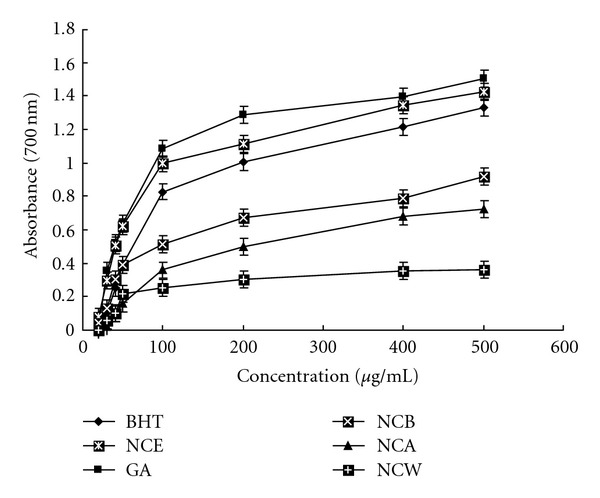
Reducing power of the extracts from *N. candida*, GA and BHT at different concentrations. Each value represents mean ± SD (*n* = 6).

**Figure 2:**
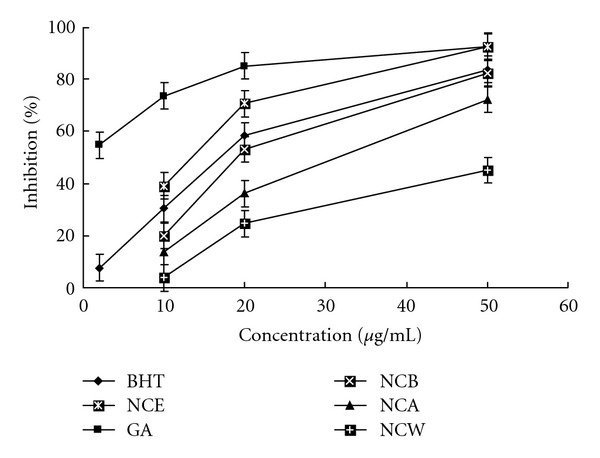
DPPH radicals scavenging activities of the extracts from N. candida, GA and BHT at different concentrations. Each value represents mean ± SD (*n* = 6).

*Nymphaea candida* Presl (or snow-white waterlily) is a herbaceous hydrophyte native to the southern Xinjiang province in China, and the flowers of *N. candida* has been used as a folk medicine for head pains, common cold, cough, hepatitis and hypertension [10]. There are 35 species from *Nymphaea* genus, and distribute widely in tropical, subtropical, temperature area [11]. Polyphenol were mainly characteristic compounds in *Nymphaea* genus [12], and these compounds were enriched by the method of ethyl acetate extracting [13]. In recent years, *N. stellata*, a folk anti-hepatitis medicine from India, has been focused much attention for its hepatoprotective effect against carbon tetrachloride-induced hepatic damage in albino rats [14], and 13 phenolic compounds were isolated from this plant [15]. However, there are no scientific studies carried out regarding antioxidant and hepatoprotective effects of *N. candida*. Hence the present study is designed to investigate the free radical scavenging and hepatoprotective activities of ethyl acetate extracts (NCE) of *N. candida* flowers against immunologic injury in primary cultured rat hepatocytes *in vitro*. NCE was selected for the studies based on the higher phenolic content and our preliminary screening tests of the extracts for their antioxidant activity. Among the extracts tested, NCE showed significantly higher activity than other extracts from *N. candida* (Figures 1 and 2).

## 2. Materials and Methods

### 2.1. Chemicals and Reagents

1,1-Diphenyl-2-picrylhydrazyl (DPPH), butylated hydroxytoluene (BHT), Collagenase (type IV), lipopolysaccharide (LPS, *E. coli* 0555:B5) and 3-(4,5-dimethylthiazol-2-yl)-2,5-diphenyltetra-zolium bromide (MTT) were purchased from Sigma Chemical Co. (St Louis, MO, USA). Dulbcco's Modifed Eagle Medium (DMEM) was purchased from Gibco Co. (Carlsbad, USA). BCG vaccine (Batch No 2007030502, expiry date 6 May 2008) was purchased from Shanghai Institute of Biological Products (Shanghai, China). Glycyrrhizin (Grz) was obtained from Chia Tai Tianqing pharmaceutical Co. Assay kits for aspartate aminotransferase (AST) and alanine aminotransferase (ALT) were provided by Zhongsheng Tech. (Beijing, China). Commercial kits used for determining nitric oxide (NO) activity were obtained from the Jiancheng Institute of Biotechnology (Nanjing, China). Other chemicals and organic solvents were of analytical grade and were purchased from a local reagent retailer.

### 2.2. Animals

Sprague-Dawley rats (male 200 ± 20 g, grade SPF, Certificate no SYXK (Xin) 2003-0001, Experimental Animal Center, Xinjiang Medical University) were used for the study. The animals were fed with a standard laboratory diet and housed in an air-conditioned room, and kept at 22  ±  1°C, 55%  ±  5% humidity with a 12 hours light/dark cycle.

### 2.3. Plant Material

The flowers of *N. candida* were collected from Hetian, XinJiang Uighur Autonomous Region, China, in August, 2005. The plant materials were identified by Researcher Yan Fu Zhang, Institute of Materia Medica of Xingjiang. A voucher specimen (no. 20050810) was deposited at the Institute of Materia Medica of Xinjiang in China.

### 2.4. Preparation of NCE and Isolation of Phenolic Compounds

The flowers were shade-dried and powdered. One kilogram of the powdered flowers was extracted with ethanol under reflux for 2 hours, and the solvent was evaporated under vacuum to afford ethanol extract (NCA). NCA was then suspended in water and successively treated with petroleum, ethyl acetate and *n*-butanol. The solvents were evaporated to afford petroleum, ethyl acetate, *n*-butanol (NCB) and aqueous residue (NCW) fractions respectively, of which the ethyl acetate fraction (NCE) was 8.6% (w/w) of starting material and was designated to be employed for the experiments. Furthermore, NCE (40 g) was chromatographed over polyamide (500 g, 30–60 mesh) with a gradient solvent system of MeOH–H_2_O (0:1–1:0). One hundred and twenty fractions were collected after combination by TLC guidance and repeated column chromatography over Sephadex LH–20 (MeOH). Finally, 13 compounds were afforded: **1** (210 mg), **2** (38 mg), **3** (61 mg), **4** (19 mg), **5** (21 mg), **6** (11 mg), **7** (8 mg), **8** (19 mg), **9** (10 mg), **10** (20 mg), **11** (10 mg), **12** (5 mg) and **13** (9 mg), respectively.

### 2.5. Phenolic Content

Total phenolic content in the extracts was determined using methods as described procedure [16] with slight modification. One millilitre of the extract was added to 2.0 mL of 0.3% sodium dodecyl sulfate and 1.0 mL of mixture with 0.6% ferric chloride–0.9% ferricyanide (1 : 0.9). The mixture was then allowed to stand for 5 minutes and 0.1 mL of 0.1 M hydrochloride acid was added for calibration, and placed 20 minutes in dark. The absorbance was measured at 720 nm in a spectrophotometer. Quantitation was based on the standard curve of gallic acid (0–1.0 mg/mL), dissolved in methanol/water (60 : 40, v/v; 0.3% HCl). Phenolic content was calculated with Gallic acid as the standard and expressed as milligrammes of gallic acid equivalent (GAE) (Table 1).

### 2.6. Evaluation of Antioxidant Activities

#### 2.6.1. Reducing Power

The reducing power of NCE was determined by the method of Yen et al. [17]. NCE, gallic acid and BHT (0.02–0.5 mg, resp.) in 1.0 mL of methanol were mixed with phosphate buffer (2.5 mL, 0.2 M, pH 6.6) and potassium ferricyanide [K_3_Fe(CN)_6_] (2.5 mL, 10 g/L; the mixture was incubated at 50°C for 20 minutes. A portion (2.5 mL) of trichloroacetic acid (100 g/L was added to the mixture, which was then centrifuged at 3000 rpm for 10 minutes. The upper layer of the solution (2.5 mL) was mixed with distilled water (2.5 mL) and FeCl_3_ (0.5 mL, 1.0 g/L) and the absorbance were measured at 700 nm. Increased absorbance of the reaction mixture indicated increased reducing power. Gallic acid (GA) and BHT were used as positive control.

#### 2.6.2. DPPH Radical Scavenging Activity Assay

DPPH free radical scavenging activity was measured according to the previously described procedure [18] with slight modification on the basis of the method of Blois [19]. Different concentrations of ethanol dilutions of samples were mixed with 2.0 vols of 6.5 × 10^−5^ M solution of DPPH. The resulting solutions were thoroughly mixed and absorbance was measured at 517 nm after keeping the tubes in dark for 30 minutes. The scavenging activity was determined by comparing the absorbance with that of control containing equal volumes of DPPH solution and ethanol. The radical scavenging activity was obtained by the following equation:

(1) $$\text{Radical}\;\text{ scavening activity (\%)}=[\frac{A_{\text{control}}-A_{\text{sample}}}{A_{\text{control}}}]\times 100.$$

The IC_50_ was defined as the concentration (in *μ*g/mL) of the extract required to deplete the amount of DPPH radical by 50%. GA and BHT were used as positive control.

#### 2.6.3. Superoxide Anion Radical (O_2_^−^) Scavenging Activity Assay

The superoxide anion radicals scavenging effect of NCE was assessed spectrophotometrically as reported previously [20]. The reaction system comprising of 0.75 mL of phenazine methosulphate (PMS, 120 *μ*M), NADH (936 *μ*M) and nitroblue tetrazolium (NBT, 300 *μ*M) in phosphate buffer (0.1 M, pH 7.4) respectively, 0.3 mL extract solution in distilled water was added subsequently. The mixture was incubated at 25°C for 5 minutes, the absorbance was read at 560 nm against blank samples. GA and BHT were used as the positive control. The percent inhibition of superoxide anion generation was calculated by the following formula:

(2) $$\text{Inhibition (\%)}\;\;=[\frac{A_{\text{control}}-A_{\text{sample}}}{A_{\text{control}}}]\times 100.$$

The IC_50_ was defined as the concentration (in *μ*g/mL) of the extract required to deplete the amount of O_2_^−^ by 50%.

#### 2.6.4. Hydroxyl Radicals (*·*OH) Scavenging Activity Assay

Scavenging of *·*OH was determined by the method of Chung et al. [21]. OH radicals were generated by incubating the following reagents in a final volume of 5.0 mL 20 *μ*M KH_2_PO_4_–KOH buffer (pH 7.4) at 37°C for 60 minutes: 0.15 mL H_2_O_2_ (10 mM), 0.15 mL Fe(NH_4_)_2_(SO_4_)_2_–EDTA (10 mM) and 0.15 mL deoxyribose (10 mM), 4.0 mL deionized water, and 0.1 mL extract solution. Degradation of deoxyribose sugar induced by *·*OH was determined by addition of 0.75 mL TBA (1% w/w) and 0.75 mL TCA (2.8% w/w) and heating at 100°C for 15 minutes. The pink chromogen formed was determined by measuring its absorbance at 536 nm. The scavenging activity on hydroxyl radical was expressed as:

(3) $$\text{Scavening activity (\%)}=[\frac{A_{\text{control}}-A_{\text{sample}}}{A_{\text{control}}}]\times 100.$$

The IC_50_ was defined as the concentration (in *μ*g/mL) of the extract required to deplete the amount of *·*OH radical by 50%. GA and BHT were used as positive control.

#### 2.6.5. Hydrogen Peroxide Radicals (H_2_O_2_) Scavenging Activity Assay

Hydrogen peroxide scavenging activity of NCE and standards was assayed by the method of Zhao et al. [22]. H_2_O_2_ (1.0 mL, 0.1 mM) and 1.0 mL of various concentrations of the extract were mixed, followed by 100 *μ*L 3% ammonium molybdate, 10 mL H_2_SO_4_ (2 M) and 7.0 mL KI (1.8 M). The mixed solution was titrated with Na_2_S_2_O_3_ (5 mM) until the yellow color disappeared. The percentage scavenging effect was calculated as

(4) $$\text{Scavening rate (\%)}=[\frac{V_{0}-V_{1}}{V_{0}}]\times 100,$$

where *V*_0_ was volume of Na_2_S_2_O_3_ solution used to titrate the control sample in the presence of hydrogen peroxide (without extract), *V*_1_ was the volume of Na_2_S_2_O_3_ solution used in the presence of NCE. The IC_50_ was defined as the concentration (in *μ*g/mL) of the extract required to deplete the amount of H_2_O_2_ radical by 50%. GA and BHT were used as positive control.

### 2.7. Evaluation of Hetapoprotective Activities

#### 2.7.1. Isolation and Culture of Primary Hepatocytes

Six rats randomly divided into two groups, and treated with or without BCG. Hepatocytes were isolated respectively from these rats by the in situ two-step collagenase perfusion technique [23, 24]. The isolated hepatocytes were counted by hemocytometer. The viability of cells was measured by trypan blue exclusion technique [25]. Cells were only used when the viability at the beginning of the experiments was more than 95%.

#### 2.7.2. Cytotoxic Assay

Cytotoxic assay was determined by a colorimetric MTT assay as described by Mosmann [26]. Hepatocytes were cultured in DMEM, and 100 *μ*L cell suspensions were plated in 96-well microtiter plates. After 16 hours of incubation at 37°C under 5% CO_2_ to allow cell attachment, the cells were treated with varying concentrations of test specimens (5–200 *μ*g/mL Grz and NCE) in DMEM (200 *μ*L) and incubated for 96 hours under the same conditions as above. After 4 hours of the addition of MTT, the medium was removed, and the blue formazan crystals that had formed were dissolved in 150 *μ*L dimethyl sulfoxide. The optical density of formazan generated from MTT was measured at 490 nm using an ELISA plate reader and the 50% inhibitory concentration (TC_50_) on cells was calculated by MTT assay.

#### 2.7.3. BCG Plus LPS-Induced Hepatocyte Injury

According to the method by krao et al. [24], hepatocytes were incubated in 24-well plate at a density of 2.5 × 10^5^ cells/well under the condition of 95% O_2_ with 5% CO_2_ after 16 hours, the plating medium was replaced by the fresh dexamethasone-free medium and then treated with LPS 10 mg/L to cause the injury of hepatocytes pretreated with BCG *in vivo*. Simultaneously, Grz 5, 10, 20 *μ*g/mL and NCE 5, 20, 80 *μ*g/mL were co-incubated with hepatocytes, respectively. After 3, 6, 12 and 24 hours, supernatants were collected to measure the biochemical index.

#### 2.7.4. Biochemical Assays

Biochemical parameters, such as the activities of aspartate transaminase (AST) and alanine transferase (ALT) in supernatant were measured spectrophotometrically using a Beckman 700 autoanalyzer with rate mode. The content of nitric oxide (NO) in supernatant was measured using a curve calibrated on sodium nitrite standard by Griess reaction [27].

### 2.8. Statistical Analysis

Data were expressed as mean ± SD and were carried out using SPSS Version 12.0 software. Analysis of variance was performed by ANOVA procedures and *P* < .05 was considered to be statistically significant.

## 3. Results

### 3.1. Total Phenolic Content

With respect to the four fractions obtained, the greatest amount of phenolic compounds was found in the EA fraction (NCE) with the value of 25.7 ± 2.1 g/100 g total phenolic expressed as gallic aicd equivalent (GAE, g/100 g of GAE). The lowest amount of phenolic compounds was measured in the aqueous residue, which was only presented as 0.38 ± 1 g/100 g of GAE.

### 3.2. Phenolic Compounds of NCE

The structures of 13 isolates were elucidated as gallic acid (**1**), gallic acid methyl ester (**2**), *p*-digalloyl acid and *m*-digalloyl acid (**3**), quercetin (**4**), kaempferol (**5**), quercetin 3-methyl ether (**6**), tricin 7-methyl ether (**7**), astragalin (**8**), quercetin 3-methyl ether 3′-*O*-xyloside (**9**), quercetin 3′-*O*-xyloside (**10**), isoquercitrin (**11**), rutin (**12**) and kaempferol-3-*O*-rutinoside (**13**) respectively, by interpretation of the spectral data (UV, IR, MS and NMR) as well as by comparison of the reported data.

### 3.3. Reducing Power of NCE

Figure 1 shows the dose-response curves for the reducing powers of the extracts from *N. candida* flower. All of the concentrations of the extracts showed higher activities than did the control and these differences were statistically significant (*P* < .01). Reducing power of NCE increased from 0.077 ± 0.006 at 20 *μ*g/mL to 1.42 ± 0.029 at 500 *μ*g/mL. At a dosage of 500 *μ*g/mL, the reducing power was significantly higher than BHT (1.33 ± 0.02) and almost equal to that of GA (1.50 ± 0.013 *μ*g/mL). Reducing power of various extracts and standard compounds followed the order: GA > NCE > BHT > NCB > NCA > NCW.

### 3.4. DPPH Radical Scavenging Activity

DPPH assay is a preliminary test to investigate the antioxidant potential of extracts.  Figure 2 shows the dose response curves of DPPH radical scavenging activities of the extracts from *N. candida*. All the extracts were capable of scavenging DPPH radicals in a concentration-dependent manner. The highest percent DPPH radical-scavenging activities were observed in the EA fraction (NCE), whilst the other samples, including the ethanol extract, *n*-butanol fractions and aqueous residue, showed lower scavenging activity. At a concentration range of 10–50 *μ*g/mL, percent inhibition and the IC_50_ of NCE were 39%–93% and 12.60 *μ*g/mL, respectively. BHT as reference drug scavenged the DPPH radical by inhibitions of 30–84% and the IC_50_ value of 15.49 *μ*g/mL. The scavenging capacity of GA (IC_50_, 1.78 *μ*g/mL) was stronger than the two former. This result suggested that NCE is a fairly good scavenger for DPPH radicals.  Table 2 shows the DPPH radical-scavenging activities of 13 compounds isolated from the EA fraction, and IC_50_ value of these compounds were showed in Table 2, respectively, of which compounds **1**–**7** and **11** showed higher activities than did NCE itself.

### 3.5. O_2_^−^, *·*OH and H_2_O_2_ Radicals Scavenging Activity of NCE

NCE significantly and dose-dependently scavenging O_2_^−^ as well and its IC_50_ value was 59.32 *μ*g/mL. The superoxide radical scavenging activity of NCE was stronger than that of BHT and weak than that of GA (Table 3). NCE also scavenged *·*OH radicals significantly (Table 3). The *·*OH radicals were generated by Fenton-type reaction and measured by their ability to degrade deoxyribose sugar into the fragments that react with thiobarbituric acid to form a pink chromogen. At a concentration of 50 *μ*g/mL, NCE could scavenge 59.02% of *·*OH radicals, IC_50_ being 24.28 *μ*g/mL. Ability of NCE to scavenge H_2_O_2_ was determined and NCE was found to dose dependently scavenge H_2_O_2_ as well (IC_50_ 86.45 *μ*g/mL) (Table 3).

### 3.6. Protective Effect of NCE on BCG Plus LPS-Induced Hepatocyte Injury

The cytotoxicity of NCE and Grz towards neonatal rat primary hepatocytes was tested. The result showed that NCE concentrations of 5–200 *μ*g/mL were almost nontoxic to the cells with TC50 value of 393.65 *μ*g/mL (Table 4).

In control group, supernatant AST and ALT activities had no significant change in 24 hours. However, in BCG combined LPS treatment group, supernatant AST and ALT activities were elevated in a time-dependent manner (*P* < .01), and with the maximum values at 12 hours. The enhancements of supernatant AST and ALT activities induced by BCG combined LPS treatment were all prevented by Grz and NCE at different time, respectively (*P* < .01) (Tables 5 and 6). In control group, NO production detected during 24 hours was <3 *μ*mo1/L. BCG combined LPS treatment group showed a higher production of NO (>5 *μ*mol/L), and the highest values were reached at 12 h. The NO generation induced by BCG combined LPS treatment was prevented by Grz within 24 hours (*P* < .01). After NCE treatment, the NO generation was showed similar result with Grz, and during all the courses was decreased significantly (*P* < .01) (Table 7).

## 4. Discussion

NCE was found to be particularly rich in polyphenols and exhibited a high reducing power, and both parameters indicated the extract to possess potent antioxidant activity. The constituents of NCE are similar to that of *N. stellata*, which has been evidenced to have hepatoprotective, and both have higher content phenolic acid, such as gallic acid and gallic acid methyl ester [14, 15]. The present study tested the ability of NCE to scavenge various free radicals. Since DPPH is known to abstract labile hydrogen and the scavenging DPPH radical ability is related to the inhibition of lipid peroxidation, it has been used to screen the antioxidant action of various compounds [28, 29]. NCE potently scavenged DPPH radicals and its scavenging activity was almost equivalent to BHT. Moreover, NCE exhibited scavenging ability for superoxide anion, hydroxyl radicals and hydrogen peroxide also. Nitric oxide is an inorganic reactive nitrogen species (RNS) synthesized in the liver by different NO synthase (NOS) isoforms, and currently considered as a fundamental intercellular and intracellular signaling molecule that is essential for the maintenance of homeostasis [30], acting either as a cytoprotective mediator or as an inducer of apoptosis [31, 32]. In the present study, there were not different distinctly on NO productions of the control group in varied time, but those increased significantly in model group. Contents of NO in NCE groups were significantly lower than that of model group. ROS, RNS and the products of their interaction are highly reactive and capable of modulating the structure and function of various cellular components [33]. Therefore, NCE, with its potent free radical quenching capacity, was expected to inhibit oxidative damage to biomolecules.

Isolated hepatocytes have the ability to retain many of the essential properties of the intact tissue, including similar permeability characteristics. The approach becomes significant because this experimental model has already proved to be a valuable tool for such studies [34]. Prior to the investigation of hepatoprotective activity for NCE, the cytotoxicity against primarily cultured rat hepatocytes was determined. The corresponding TC_50_ values of NCE and Grz on hepatocytes were all >80 *μ*g/mL, and thus the studied samples were considered as non-cytotoxic to the cell line. BCG activates and sensitizes T lymphocyte, especially sensitizing macrophage cells and Kupffer cells. After injected LPS, macrophage cells were further activated, and released various cytokines that made hepatic injury, such as NO, free radical and TNF [35–37]. Thus, BCG plus LPS can induce the sensitive immunological response, which will lead to leakage of liver cells. Large amount of ALT and AST released to supernatants is signal of liver cell leakage. In immunological injury of primary cultured rat hepatocytes, levels of ALT and AST in NCE groups (5, 20 and 80 *μ*g/mL dosage) were significantly lower than that of model group. The effect of NCE at the dosage of 80 *μ*g/mL was comparable with that of Grz at 20 *μ*g/mL. NCE probably decreased the amount of ALT and AST in supernatants by suppressing the immunity reaction and reducing the leakage of liver cells. Therefore, it may be concluded that NCE has protective activity on immunological injury *in vitro*.

In conclusion, our current investigation verifies the hepatoprotective and antioxidative effects of NCE *in vitro*. The scavenging of active radical may be one of main mechanisms of protective of NCE against BCG plus LPS-induced cytotoxicity in primary cultured rat hepatocytes. This evidence provides a scientific explanation for the folkloric uses of *N. candida* in the treatment of hepatitis, and the further experiment *in vivo* will be carried out for studying its effective mechanism.

## Funding

This work was financially supported by the opening foundation of Xinjiang Key Laboratory for Research and Development of Uighur Medicine (XJYS0207-2005-01).

## Figures and Tables

**Table 1:** Total phenolic content of *N. candida* extracts.

	Ethanol extracts (NCA)	Ethyl acetate Fr. (NCE)	*n*-BuOH Fr. (NCB)	Aqueous residue Fr. (NCW)

Total phenolic content (g/100 g)	7.52 ± 1.8	25.7 ± 2.1	12.11 ± 1.29	0.38 ± 1

**Table 2:** DPPH radical scavenging activities of 13 phenolic compounds from NCE.

Compounds	IC_50_ (*μ*g/mL)
Gallic acid (**1**)	1.78
Gallic acid methyl ester (**2**)	2.21
*p*-Digalloyl acid and *m*-digalloyl acid (**3**)	2.53
Quercetin (**4**)	1.97
Kaempferol (**5**)	5.56
Quercetin 3-methyl ether (**6**)	4.78
Tricin 7-methyl ether (**7**)	6.26
Astragalin (**8**)	63.53
Quercetin 3-methyl ether 3′*-O*-xyloside (**9**)	28.20
Quercetin 3′-*O*-xyloside (**10**)	20.11
Isoquercitrin (**11**)	9.42
Rutin (**12**)	16.62
Kaempferol - 3-*O*-rutinoside (**13**)	81.22

**Table 3:** Scavenging of, superoxide (O_2_^−^), hydroxyl radical (*·*OH) and hydrogen peroxide (H_2_O_2_) by NCE.

Group	Concentration	O_2_^−^		*·*HO		H_2_O_2_	
	(*μ*g/mL)	Inhibition (%)	IC_50_ (*μ*g/mL)	Inhibition (%)	IC_50_ (*μ*g/mL)	Inhibition (%)	IC_50_ (*μ*g/mL)

Gallic acid	2	9.05 ± 0.10		30.56 ± 1.63		11.12 ± 1.12	
	10	16.95 ± 0.34	29.39	43.2 ± 0.53	16.29	20.8 ± 1.54	31.87
	50	68.72 ± 0.25		62.14 ± 0.74		63.7 ± 1.28	

BHT	2	5.57 ± 0.47		20.85 ± 1.02		4.42 ± 0.12	
	10	23.12 ± 1.32	71.87	31.2 ± 0.44	83.56	18.29 ± 0.29	131.6
	50	39.83 ± 1.21		45.69 ± 1.89		30.43 ± 0.31	

NCE	10	11.6 ± 0.70		25.43 ± 0.22		19.02 ± 0.64	
	20	25.01 ± 1.01	59.32	38.22 ± 0.96	24.28	27.22 ± 1.49	86.45
	50	44.16 ± 0.43		59.02 ± 1.16		40.91 ± 1.26	

IC_50_, value of the 50% inhibition concentration; inhibition (%), percent inhibition of means of six replicates from the control.

**Table 4:** Cytotoxicity of NCE and Grz against primarily cultured rat hepatocytes.

Group	Concentration (*μ*g/mL)	Inhibition (%)	TC_50_ (*μ*g/mL)
Grz	200	29.12 ± 0.02	465.12
	100	16.19 ± 0.06	
	25	4.21 ± 0.08	
	5	0.86 ± 0.06	

NCE	200	40.12 ± 0.07	393.65
	100	19.83 ± 0.06	
	25	3.26 ± 0.21	
	5	1.60 ± 0.1	

TC_50_, value of the 50% inhibition concentration (%); inhibition (%), percent inhibition of means of six replicates from the control.

**Table 5:** Effect of NCE on supernatant AST levels in immunologic injury in primary cultured rat hepatocytes *in vitro*.

Group	Dose	AST (U/L)			
	*μ*g/mL	3 hours	6 hours	12 hours	24 hours

Control	—	21.45 ± 2.92	24.17 ± 3.21	40.87 ± 6.01	41.00 ± 8.07
BCG + LPS	—	57.03 ± 2.88^a^	90.55 ± 10.46^a^	101.88 ± 10.71^a^	63.34 ± 8.90^a^
BCG + LPS + Grz	5	65.15 ± 16.64	59.89 ± 8.66^b^	73.84 ± 10.89^b^	37.67 ± 6.16^b^
	10	48.53 ± 10.34	43.55 ± 12.02^b^	60.66 ± 6.05^b^	41.12 ± 16.37^b^
	20	57.34 ± 9.57	59.68 ± 16.59^b^	55.06 ± 8.17^b,c^	33.38 ± 7.92^b^
BCG + LPS + NCE	5	67.78 ± 6.41	53.79 ± 8.83^b^	58.66 ± 4.72^b^	37.76 ± 3.01^b^
	20	56.88 ± 13.46	55.55 ± 7.20^b,d^	48.72 ± 2.10^b^	35.43 ± 4.34^b^
	80	57.65 ± 6.24	36.71 ± 10.00^b,d^	49.94 ± 6.25^b^	35.63 ± 10.00^b^

Values are the mean ± SD, *n* = 6. ^a^ *P* < .01 compared with control group; ^b^ *P* < .01 compared with BCG + LPS group; ^c^ *P* < .05 compared with BCG + LPS + Grz group (5 *μ*g/mL); ^d^ *P* < .05 compared with BCG + LPS + NCE group (5 *μ*g/mL).

**Table 6:** Effect of NCE on supernatant ALT levels in immunologic injury in primary cultured rat hepatocytes *in vitro*.

Group	Dose	ALT (U/L)			
	*μ*g/mL	3 hours	6 hours	12 hours	24 hours

Control	—	3.16 ± 0.58	3.68 ± 0.42	4.68 ± 0.31	3.65 ± 0.53
BCG + LPS	—	5.69 ± 0.91^a^	7.68 ± 0.74^a^	9.43 ± 1.23^a^	6.58 ± 0.83^a^
BCG + LPS + Grz	5	5.12 ± 1.36	4.08 ± 1.00^b^	6.61 ± 1.01^b^	3.11 ± 0.59^b^
	10	3.62 ± 0.73^b,e^	3.29 ± 1.05^b^	5.14 ± 0.85^b,e^	3.80 ± 1.42^b^
	20	3.98 ± 0.64^b^	4.80 ± 1.65^b^	4.87 ± 0.80^b,d^	3.20 ± 1.24^b^
BCG + LPS + NCE	5	5.71 ± 1.10	3.95 ± 0.53^b^	6.06 ± 0.70^b^	3.52 ± 0.37^b^
	20	4.25 ± 1.17^c^	3.77 ± 0.63^b^	4.88 ± 0.30^b^	3.21 ± 0.49^b^
	80	4.71 ± 1.23	2.53 ± 0.97^b^	4.07 ± 0.86^b^	3.29 ± 0.98^b^

Values are the mean ± SD, *n* = 6. ^a^ *P* < .01 compared with control group; ^b^ *P* < .01 and ^c^ *P* < .05 compared with BCG + LPS group; ^d^ *P* < .01 and ^e^ *P* < .01 compared with BCG + LPS + Grz group (5 *μ*g/mL).

**Table 7:** Effect of NCE on supernatant NO production in immunologic injury in primary cultured rat hepatocytes *in vitro*.

Group	Dose	NO (*μ*mol/l)			
	*μ*g/mL	3 hours	6 hours	12 hours	24 hours

Control	—	2.38 ± 0.19	2.52 ± 0.18	2.80 ± 0.21	2.82 ± 0.21
BCG + LPS	—	5.03 ± 0.33^a^	8.51 ± 0.49^a^	10.57 ± 0.77^a^	7.41 ± 0.77^a^
BCG + LPS + Grz	5	4.96 ± 0.31	6.49 ± 0.74^b^	8.13 ± 0.55^b^	5.73 ± 0.64^b^
	10	4.25 ± 0.43^b,c^	5.83 ± 0.48^b,c^	7.65 ± 0.55^b,c^	4.84 ± 0.4^b,d^
	20	3.63 ± 0.38^b,c^	4.83 ± 0.55^b,c^	6.08 ± 0.39^b,c^	4.64 ± 0.51^b,c^
BCG + LPS + NCE	5	5.10 ± 0.13	6.41 ± 0.73^b^	7.89 ± 0.39^b^	5.87 ± 0.45^b^
	20	4.53 ± 0.73	5.84 ± 0.51^b^	7.41 ± 0.71^b^	5.50 ± 0.58^b^
	80	4.44 ± 0.7	5.07 ± 0.60^b,e^	6.69 ± 0.39^b,e^	5.08 ± 0.69^b,f^

Values are the mean ± SD, *n* = 6. ^a^ *P* < .01 compared with control group; ^b^ *P* < .01 compared with BCG + LPS group; ^c^ *P* < .05; ^d^ *P* < .01 compared with BCG + LPS + Grz group (5 *μ*g/mL); ^e^ *P* < .05 and ^f^ *P* < .01 compared with BCG + LPS + NCE group (5 *μ*g/mL).
